# Comparison of two different frailty measurements and risk of hospitalisation or death from COVID-19: findings from UK Biobank

**DOI:** 10.1186/s12916-020-01822-4

**Published:** 2020-11-10

**Authors:** Fanny Petermann-Rocha, Peter Hanlon, Stuart R. Gray, Paul Welsh, Jason M. R. Gill, Hamish Foster, S. Vittal Katikireddi, Donald Lyall, Daniel F. Mackay, Catherine A. O’Donnell, Naveed Sattar, Barbara I. Nicholl, Jill P. Pell, Bhautesh D. Jani, Frederick K. Ho, Frances S. Mair, Carlos Celis-Morales

**Affiliations:** 1 Institute of Health and Wellbeing, University of Glasgow, Glasgow, UK; 2 British Heart Foundation Glasgow Cardiovascular Research Centre, Institute of Cardiovascular and Medical Sciences, College of Medical, Veterinary and Life Sciences, University of Glasgow, Glasgow, G12 8TA UK; 3 Centre of Exercise Physiology Research (CIFE), Universidad Mayor, Santiago, Chile; 4 Laboratorio de Rendimiento Humano, Grupo de Estudio en Educación, Actividad Física y Salud (GEEAFyS), Universidad Católica del Maule, Talca, Chile

**Keywords:** COVID-19, Coronavirus, Frailty, Risk factors

## Abstract

**Background:**

Frailty has been associated with worse prognosis following COVID-19 infection. While several studies have reported the association between frailty and COVID-19 mortality or length of hospital stay, there have been no community-based studies on the association between frailty and risk of severe infection. Considering that different definitions have been identified to assess frailty, this study aimed to compare the association between frailty and severe COVID-19 infection in UK Biobank using two frailty classifications: the frailty phenotype and the frailty index.

**Methods:**

A total of 383,845 UK Biobank participants recruited 2006–2010 in England (211,310 [55.1%] women, baseline age 37–73 years) were included. COVID-19 test data were provided by Public Health England (available up to 28 June 2020). An adapted version of the frailty phenotype derived by Fried et al. was used to define frailty phenotype (robust, pre-frail, or frail). A previously validated frailty index was derived from 49 self-reported questionnaire items related to health, disease and disability, and mental wellbeing (robust, mild frailty, and moderate/severe frailty). Both classifications were derived from baseline data (2006–2010). Poisson regression models with robust standard errors were used to analyse the associations between both frailty classifications and severe COVID-19 infection (resulting in hospital admission or death), adjusted for sociodemographic and lifestyle factors.

**Results:**

Of UK Biobank participants included, 802 were admitted to hospital with and/or died from COVID19 (323 deaths and 479 hospitalisations). After analyses were adjusted for sociodemographic and lifestyle factors, a higher risk of COVID-19 was observed for pre-frail (risk ratio (RR) 1.47 [95% CI 1.26; 1.71]) and frail (RR 2.66 [95% CI 2.04; 3.47]) individuals compared to those classified as robust using the frailty phenotype. Similar results were observed when the frailty index was used (RR mildly frail 1.46 [95% CI 1.26; 1.71] and RR moderate/severe frailty 2.43 [95% CI 1.91; 3.10]).

**Conclusions:**

Frailty was associated with a higher risk of severe COVID-19 infection resulting in hospital admission or death, irrespective of how it was measured and independent of sociodemographic and lifestyle factors. Public health strategies need to consider the additional risk that COVID-19 poses in individuals with frailty, including which additional preventive measures might be required.

## Background

Since January 2020, COVID-19—the disease generated by the severe acute respiratory syndrome coronavirus 2 (SARS-CoV-2)—has reached pandemic status due to its infectivity and fatality [1, 2]. Globally, more than 43 million people have been infected with the virus, and more than 1 million have died from it up to the end of October 2020 [3]. Age, sex, ethnicity, and the pre-existence of multiple comorbidities have been recognised as factors associated with prognosis in COVID-19 [1, 4, 5]. Frailty is also common among hospital inpatients with COVID-19 [6–8].

Frailty is a clinical state associated with older age and characterised by an increased susceptibility to decompensation in response to physiological stress [9]. While a large number of measures have been used to identify frailty, two operational definitions of frailty have dominated the scientific literature on this field: the frailty phenotype and the frailty index [10]. Using each of these definitions, frailty has been associated with higher risk of disability, morbidity, and mortality [11]. Several studies have also reported a high prevalence of frailty in people with chronic respiratory diseases [12–14], suggesting that frailty may be an independent risk factor in the development and progression of respiratory diseases [14].

One in four adults older than 85 years lives with frailty, and according to a recent systematic review and meta-analysis, one in six community-dwelling older adults might have frailty [15]. Frailty is not, however, only associated with older age. Frailty and pre-frailty are higher among those living with socioeconomic deprivation and those with multimorbidity (≥ 2 long-term conditions [LTCs]) even in middle-age [16].

During this pandemic, the clinical importance of frailty is highlighted in clinical guidelines recommending frailty assessment for all adults admitted to hospital [17, 18]. In this context, frailty assessment is recommended as part of a holistic approach to identifying patients in need of comprehensive geriatric assessment as well as identifying people with the most severe frailty to guide consideration of the appropriateness of critical care interventions. The literature around COVID-19 and frailty is rapidly evolving, and a number of hospital-based studies have demonstrated that frailty is associated with greater risk of mortality and intensive-care admission with COVID-19 [7, 8, 19–21]. Most notably, the multi-centre COVID-19 in Older People (COPE) study demonstrated that the Clinical Frailty Scale was a better predictor of in-hospital death than either age or comorbidity [19]. However, this association with in-hospital mortality has not been consistently observed across all studies to-date [7]. To our knowledge, there have been no community-based studies on the association between frailty and risk of COVID-19 infection. Therefore, this study aimed to compare the association between frailty and severe COVID-19 infection resulting in hospital admission or death in UK Biobank using two different approaches to measuring frailty: the frailty phenotype and the frailty index.

## Methods

This study uses data from UK Biobank. Over 500,000 participants (5.5% response rate), aged 37 to 73 years from the general population, were recruited into UK Biobank between March 2006 and December 2010 [22]. Participants attended one of 22 assessment centres across the UK [23, 24] where they completed a touch-screen questionnaire, had physical measurements taken, and provided biological samples, as described in detail elsewhere [23, 24]. For this study, only participants from English assessment centres were included since data on COVID-19 status of UK Biobank participants were only provided from Public Health England (PHE) and no other parts of the UK. Additionally, we excluded all participants known to have died of non-COVID causes up to 16 March 2020.

### Outcomes

PHE provided the COVID-19 test data, including the specimen date, location, and result (positive or negative) of the test. Data were available for the period 16 March 2020 to 28 June 2020. Records were also linked to inpatient Hospital Episode Statistics and national mortality registers. From these, we identified individuals who tested positive during an inpatient hospital episode, in the 14 days prior to admission, or within 7 days of hospital discharge. We also identified individuals who had died with COVID-19 (ICD-10 code U70 on death certification). Participants meeting this definition were considered to have ‘severe COVID-19’ leading to hospitalisation or death. We compared these participants to those who were alive during the pandemic but who had not had an admission to hospital associated with COVID-19.

More information on COVID-19 in UK Biobank can be found here: http://biobank.ndph.ox.ac.uk/ukb/exinfo.cgi?src=COVID19_tests.

### Exposures: the frailty phenotype and the frailty index

We undertook our analyses using two different approaches to assessing frailty: the frailty phenotype and the frailty index. Both frailty assessments were based on assessment centre data collected at baseline (2006–2010).

An adapted version of the frailty phenotype derived by Fried et al., and previously published using the UK Biobank baseline data, was used in this study [16]. The Fried phenotype uses the following five criteria: weight loss, exhaustion, physical activity, walking speed, and grip strength [25]. Some of these criteria were adapted to fit the data available within UK Biobank [16].

Weight loss was derived from self-report of weight loss in the previous year, dichotomised into yes or no (same weight or gained weight). Exhaustion was derived from the self-report of tiredness in the last 2 weeks categorised as follows: not at all, several days, more than half the days, and nearly every day. Those participants who reported tiredness more than half the days or nearly every day were identified as meeting the Fried criterion for exhaustion. Walking pace was categorised as slow, average, or brisk. To derive a proxy for gait speed, this was then dichotomised into slow or normal (average or brisk pace). Grip strength was measured using a Jamar J00105 hydraulic hand dynamometer. Isometric grip force was assessed from a single 3-s maximal grip effort, separately in the right and left arms, with the participant seated upright with their elbow by their side and flexed at 90° so that their forearm was facing forwards and resting on an armrest. The average of the right and left values were expressed in absolute units (kg) and used in subsequent analyses. Low grip strength was based on cut-offs from Fried et al.’s original description, stratified by sex and body mass index. Physical activity was self-reported and classified as follows: none (response: none or light activity with a frequency of once per week or less = 1) and physically active (medium or heavy activity, or light activity more than once per week = 0) [16].

Participants were classified as frail if they fulfilled three or more criteria, pre-frail if they fulfilled one or two criteria, and robust if they did not fulfil any criteria at baseline. The three frailty groups were mutually exclusive.

A frailty index has previously been validated using baseline data from UK Biobank [26]. The frailty index approach was developed by Rockwood and Mitnitski and is a cumulative count of ‘deficits’ [27, 28]. The frailty index was initially described using 70 deficits from the Canadian Study of Health Ageing [28]. However, the frailty index method was developed as a standard technique which can be adapted to the deficits available in a given dataset [29]. The adaptation of the frailty index approach to UK Biobank is described in detail elsewhere [26]. Briefly, deficits should be associated with age, associated with poor health status, and be neither universal nor too rare within the target population [29]. A frailty index is calculated for each individual by calculating the total number of deficits present in an individual and divided by the total number of possible deficits measurable to give a value between 0 and 1 (higher values indicating a greater degree of frailty). We applied a previously validated frailty index comprising 49 self-reported questionnaire items related to health, presence of disease and disability, and mental wellbeing [26]. Based on this frailty index, we classified participants as being robust (frailty index < 0.12), mildly frail (frailty index 0.12–0.24), or moderate/severely frailty (frailty index > 0.24) [30].

### Covariates

Age at baseline was calculated from dates of birth and baseline assessment. Current age was derived from dates of birth and last data from COVID-19 assessment (June 2020). Area-based socioeconomic status (deprivation) was derived from the postcode of residence, using the Townsend score [31]. Ethnicity was self-reported and categorised, in this study, into white and non-white. This approach was selected due to insufficient statistical power in the non-white subgroups. Self-reported smoking status was categorised as never, former, or current smoker. Frequency of alcohol intake was self-reported at baseline via touch-screen questionnaire and categorised as never/special occasions only, 1–3 times per month, 1–4 times per week, or daily/almost daily. Prevalent morbidity was ascertained during a nurse-led interview at baseline. We calculated morbidity count based on 43 LTCs originally developed for a large epidemiological study in Scotland and subsequently adapted for UK Biobank [32, 33]. Further details of these measurements can be found in the UK Biobank online protocol (http://www.ukbiobank.ac.uk).

### Ethical approval

UK Biobank was approved by the North West Multi-Centre Research Ethics Committee (Ref: 11/NW/0382). All participants provided written informed consent to participate in the UK Biobank study. The study protocol is available online (http://www.ukbiobank.ac.uk). This work was conducted under the UK Biobank application number 14151.

### Statistical analyses

Descriptive characteristics are presented as means with standard deviations (SD) for quantitative variables and as percentages for categorical variables, broken down by each frailty classification and the presence or absence of severe COVID-19 infection (defined as hospitalisation or death with COVID-19). Poisson regression models with robust standard errors were used to analyse the associations between both the frailty phenotype and the frailty index and severe COVID-19. The results are reported as risk ratios (RRs) with their 95% confidence intervals (CIs) [34]. Poisson regression models with robust standard errors were used because they provide RR estimates, instead of odds ratios, which are easier to interpret [35].

We ran four models including an increasing number of covariates: model 1 (minimally adjusted), adjusted by age and sex; model 2, as per model 1 but also included deprivation, and white versus non-white groups; model 3, included smoking and alcohol intake only; and model 4, included all covariates in models 2 and 3. An additional sensitivity analysis (model 5) was performed aiming to investigate whether the association between the frailty phenotype and COVID-19 was explained by multimorbidity. This model included covariates in model 4, but additionally included multimorbidity (based on a count of 43 diseases and coded as ordinal 0, 1, 2, 3, and ≥ 4 LTCs). This model was carried out for the frailty phenotype only because the frailty index is partly based on the presence of morbidity. All these covariates were selected because they have been recognised as being associated with prognosis of COVID-19 as well as being associated with frailty status, and may therefore potentially confound the relationship between frailty and COVID-19 [1, 4, 5].

Finally, to investigate whether the associations between severe COVID-19 and frailty differed by subgroups, the analyses were re-run stratified by sex and age categories (based on age in June 2020: < 60, 60–70, and > 70 years). An interaction term among the subgroups, the frailty classifications, and severe COVID-19 was fitted into the regression model to test for interaction.

All statistical analyses were performed using R version 3.6.1. Only participants with full data available for both classifications and covariates were included in the analyses.

## Results

A total of 420,577 UK Biobank participants in England were eligible for inclusion, of whom 383,845 had data on both frailty phenotype and frailty index. Of these, 802 were either hospitalised with and/or died from COVID-19 and were classified as ‘severe COVID-19’ (323 deaths and 479 hospitalisations only). The proportion of people identified as frail at baseline, along with the overlap between the frailty phenotype and the frailty index, is shown in Table 1. Out of 383,845 participants, 11,836 (3.1%) participants were frail according to the frailty phenotype, and 15,958 (4.1%) had moderate or severe frailty according to the frailty index. Using the frailty phenotype, and compared with robust individuals, pre-frail and frail individuals with severe COVID-19 were older, more likely to be deprived, non-white, current smoker, to never or occasionally drink alcohol, and to have one or more morbidities (Table 2). Similar characteristics were identified when individuals with mild frailty and moderate/severe frailty were compared with robust individuals using the frailty index (Table S1).

**Table 1:** Overlap between the frailty phenotype and frailty index

	Robust, *n* (%)	Mild, *n* (%)	Moderate or severe, *n* (%)	Total
Robust, *n* (%)	170,964 (44.5)	55,456 (14.5)	2665 (0.7)	229,085 (59.7)
Pre-frail, *n* (%)	75,898 (19.8)	57,719 (15.0)	9307 (2.4)	142,924 (37.2)
Frail, *n* (%)	1770 (0.5)	6080 (1.6)	3986 (1.0)	11,836 (3.1)
Total	248,632 (64.8)	119,255 (31.1)	15,958 (4.1)	**383,845**

Data presented as absolute numbers and prevalence for each frailty measurement

**Table 2:** Characteristics of the population according to their COVID-19 test and the frailty phenotype

	No COVID-19 associated admission or death			Severe COVID-19 infection		
	Robust	Pre-frail	Frail	Robust	Pre-frail	Frail
Total, *n*	228,731	142,550	11,762	354	374	74
Baseline age (years), mean (SD)	56.0 (8.1)	56.5 (8.1)	57.4 (7.7)	60.3 (7.7)	59.8 (7.8)	59.5 (7.9)
Current age (years), mean (SD)	67.1 (8.1)	67.5 (8.1)	68.4 (7.7)	71.3 (7.8)	70.8 (7.8)	70.6 (8.0)
Sex (female), *n* (%)	120,231 (52.6)	83,011 (58.2)	7772 (66.1)	116 (32.8)	145 (38.8)	35 (47.3)
Deprivation, *n* (%)						
Lower	83,333 (36.5)	42,368 (29.7)	2086 (17.7)	92 (26.0)	87 (23.3)	9 (12.1)
Middle	78,557 (34.3)	46,199 (32.4)	2947 (25.1)	123 (34.7)	95 (25.4)	15 (20.3)
Higher	66,841 (29.2)	53,983 (37.9)	6729 (57.2)	139 (39.3)	192 (51.3)	50 (67.6)
Ethnicity, *n* (%)						
White	220,508 (96.4)	132,223 (92.8)	10,204 (86.8)	325 (91.8)	324 (86.6)	63 (85.1)
Non-white	8223 (3.6)	10,327 (7.2)	1558 (13.2)	29 (8.2)	50 (13.4)	11 (14.9)
Smoking status, *n* (%)						
Never	130,457 (57.0)	76,966 (54.0)	5737 (48.8)	134 (37.9)	177 (47.3)	25 (33.8)
Previous	79,074 (34.6)	49,909 (35.0)	4021 (34.2)	173 (48.9)	151 (40.4)	37 (50.0)
Current	19,200 (8.4)	15,675 (11.0)	2004 (17.0)	47 (13.2)	46 (12.3)	12 (16.2)
Alcohol intake, *n* (%)						
Daily or almost daily	53,467 (23.4)	25,316 (17.8)	1176 (10.0)	77 (21.8)	62 (16.6)	9 (12.2)
One to four times a week	119,836 (52.4)	66,267 (46.5)	3711 (31.6)	172 (48.6)	147 (39.3)	24 (32.4)
One to three times a month	23,582 (10.3)	17,695 (12.4)	1487 (12.6)	40 (11.2)	43 (11.5)	10 (13.5)
Never or special occasions	31,846 (13.9)	33,272 (23.3)	5388 (45.8)	65 (18.4)	122 (32.6)	31 (41.9)
Multimorbidity, *n* (%)						
None	93,868 (41.0)	40,383 (28.4)	1068 (9.1)	87 (24.6)	65 (17.4)	6 (8.1)
1	78,185 (34.2)	46,384 (32.5)	2357 (20.0)	119 (33.6)	106 (28.3)	7 (9.5)
2–3	51,407 (22.5)	46,373 (32.5)	5371 (45.7)	133 (37.6)	156 (41.7)	36 (48.6)
≥ 4	5271 (2.3)	9410 (6.6)	2966 (25.2)	15 (4.2)	47 (12.6)	25 (33.8)

The frailty phenotype was derived using an adaptation from the original derived by Fried et al. Participants were classified as frail if they fulfilled three or more criteria, pre-frail if they fulfilled one or two criteria, and robust if they did not fulfil any criteria at baseline

*SD* standard deviation, *n* number

**Fig. 1:**
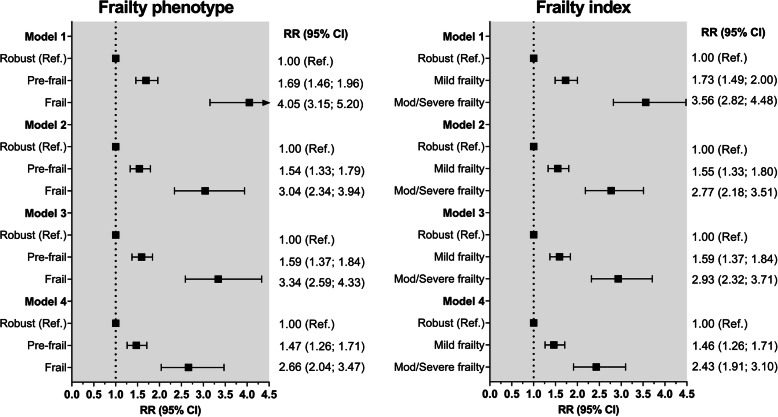
Associations between the frailty phenotype, the frailty index, and severe COVID-19 infection. Data presented as RRs with their 95% CIs using Poisson regression analyses. Robust individuals were used as the reference group for the frailty phenotype and the frailty index. Model 1, adjusted by age and sex; model 2, as model 1 but also included deprivation, and ethnicity: white versus others; model 3, included smoking and alcohol intake only; model 4, included the covariates in models 2 and 3

Associations between the frailty phenotype and the frailty index and severe COVID-19 are presented in Fig. 1. Using the frailty phenotype, and compared with non-frail individuals, being pre-frail and frail were associated with 1.69 times [95% CI 1.46; 1.96] and more than four times [RR 4.05 95% CI 3.15; 5.20] higher risk of severe COVID-19, respectively (age- and sex-adjusted model). These associations were attenuated but remained when analyses were adjusted both for sociodemographic and lifestyle factors (RR_pre-frail_ 1.47 [95% CI 1.26; 1.71] and RR_frail_ 2.66 [95% CI 2.04; 3.47]) (model 4, Fig. 1). Results were similar in analyses using the frailty index, although effect sizes were slightly smaller. In the age- and sex-adjusted model, individuals with mild frailty and moderate/severe frailty had 1.73 [95% CI 1.49; 2.00] and 3.56 [95% CI 2.82; 4.48] times higher risk of severe COVID-19, respectively, compared with robust individuals. When we further adjusted the model for sociodemographic and lifestyle factors, the associations attenuated further, but remained (RR_mild-frail_ 1.46 [95% CI 1.26; 1.71] and RR_mod/severe frail_ 2.43 [95% CI 1.91; 3.10]) (model 4, Fig. 1). In addition, when multimorbidity was included in the sensitivity analysis for the frailty phenotype only (Table S2, model 5), the associations remained but were further attenuated (RR_pre-frail_ 1.35 [95% CI 1.16; 1.57] and RR_frail_ 1.99 [95% CI 1.51; 2.62]).

For the frailty index, we repeated model 4 treating the frailty index as continuous. There was a RR of 1.53 (95% CI 1.40; 1.67) per 0.1-point increase in the frailty index.

**Fig. 2:**
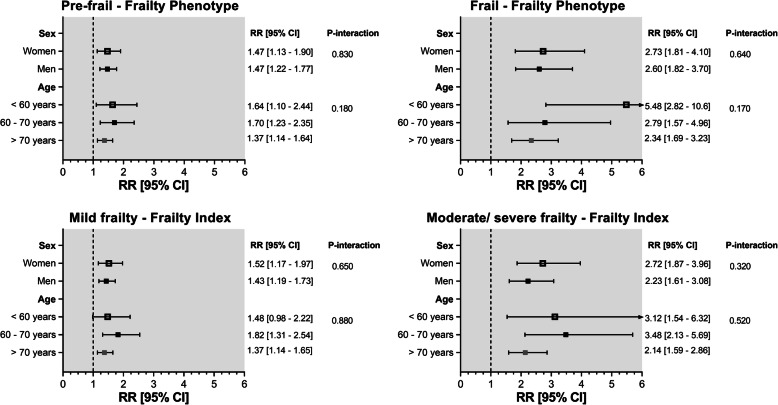
Associations between the frailty phenotype, the frailty index, and severe COVID-19 infection by subgroups. Data presented as RRs with their 95% CIs using Poisson regression analyses. Robust individuals were used as the reference group for the frailty phenotype and the frailty index. All the analyses were adjusted by age, sex, deprivation, ethnicity, smoking, and alcohol intake when these were not the subgroup used

No significant interaction was observed between either frailty definition and age or sex (Fig. 2). When the analyses were stratified by subgroups (sex and age categories), we identified that the associations were similar for both sexes and age categories using both the frailty index and the frailty phenotype (Fig. 2). However, the effect of frailty using the frailty phenotype was higher in people aged < 60 at the time of the pandemic (Fig. 2).

## Discussion

We demonstrated that people previously identified as frail were at higher risk of severe COVID-19 infection, after adjustment for sociodemographic and lifestyle factors and independent of multimorbidity in the case of the frailty phenotype. These findings were consistent using two different approaches to assessing frailty: the frailty phenotype and the frailty index.

Attenuation following adjustment for multimorbidity using the frailty phenotype is to be expected since morbidity contributes to frailty: 91.9% of frail individuals with severe COVID-19 had multimorbidity versus 75.6% of those classified as robust. On the other hand, we identified that the associations were similar after stratification by sex and age (except for some exceptions probably underpowered). The latter highlights the impact of our findings but also reinforces the implications of frailty beyond ageing [16].

Frailty (either moderate or severe) has been identified among inpatients affected by COVID-19 [6–8, 19–21]. This is consistent with previous findings where frailty has been recognised as a critical prognostic factor of viral pneumonia among inpatients [13]. However, the literature has reported heterogeneous results between frailty and COVID-19 mortality. For example, Miles et al., using data from 377 older inpatients admitted to a London hospital, identified that frailty was not associated with mortality rates after COVID-19 [7]. However, Hewitt et al. and De Smet et al., using the Clinical Frailty Scale (CFS), demonstrated that frail individuals had a higher risk of mortality after adjusting for covariates [19, 20]. Our study is novel since it demonstrates an increased risk of hospitalisation or death from COVID-19 among community-dwelling individuals, but does not investigate prognosis after hospitalisation.

As with COVID-19, frailty is strongly associated with ageing. It also shares some common modifiable risk factors with COVID-19, such as body mass index, muscle strength, respiratory function, and slow gait speed [4, 36]. Although chronological age cannot be modified, key proxies of physical function related to ageing and frailty can. There is evidence that frailty could be reversed with exercise interventions in some older adults [37]. A recent trial conducted in hospitalised frail individuals showed that an exercise intervention was effective at helping to reverse the functional decline associated with ageing [38]. Therefore, there is a need to recognise frail individuals as a higher risk group and determine how best to balance their competing risks, providing greater protection from infection through existing non-pharmaceutical interventions such as physical distancing and shielding, while encouraging and supporting greater physical activity to reduce their frailty. This could potentially be achieved through home training programmes for people with restriction of mobility [39], and perhaps drawing upon the intersection between frailty and respiratory disease [40, 41]. Of note, interventions that prevent, delay, or reverse frailty are likely to have significant public health impact beyond the COVID-19.

### Limitations

This study is not without limitations. Firstly, both the frailty phenotype and the frailty index were identified from baseline UK Biobank data (between 10 and 14 years prior to the COVID-19 pandemic). Therefore, we did not have data on subsequent frailty status. Frailty is a dynamic state and is likely to have worsened over time. Consequently, transitioning from a frail to a less frail state is relatively uncommon; however, a proportion of those not identified as frail at baseline are likely to have become frail during the follow-up [42]. Therefore, our results may be an underestimate of the magnitude of the association between frailty and COVID-19. Secondly, while the frailty phenotype and the frailty index are the most widely validated epidemiological measurements of frailty, they are not routinely used within clinical practice. NICE (National Institute for Health and Care Excellence) has recommended using the CFS for the assessment of frailty in the COVID-19 guideline [17]. However, due to the absence of some of the variables included in the CFS in the UK Biobank study, we used a frailty index [26] and an adapted version of the frailty phenotype [16]. While there appears to be a modest degree of overlap between the CFS and other frailty definitions [43], few studies have assessed in detail how the CFS related to measures such as the frailty index or frailty phenotype. The frailty phenotype was an adaptation of the original description by Fried et al. [25], and the frailty index was derived from self-reported data only. Finally, the UK Biobank study is not a nationally representative sample in terms of lifestyle, morbidity, ethnicity, and socioeconomic status [44]. This lack of representativeness is an important limitation, particularly as characteristics such as ethnicity and comorbidities appear to be strongly associated with prognosis in COVID-19 [45]. Therefore, the summary statistics should not be generalised [44], even though effect size estimates are comparable with nationally representative cohorts [46].

## Conclusion

Individuals with frailty had a higher risk of severe COVID-19 regardless of the frailty measure used. As the lockdown measures have changed during the course of the pandemic, guidance on how we can protect individuals with frailty should be considered, including whether more protective, preventive measures are required. Moreover, considering we are facing a new COVID-19 outbreak and that confinement could exacerbate frailty [47], further public health policies to minimise the risk of developing this syndrome are more urgent than ever.

## Supplementary Information

**Additional file 1: Table S1.** Characteristics of the population according to their COVID-19 test and the frailty index. **Table S2.** Associations between the frailty phenotype and severe COVID-19 infection (sensitivity analysis).

## Data Availability

All UK Biobank information is available online on the webpage www.ukbiobank. Data access is available through applications. This research was conducted using the application number 14151.

## Abbreviations

CFS: Clinical Frailty Scale
CIs: Confidence intervals
FI: Frailty index
LTCs: Long-term conditions
NICE: National Institute for Health and Care Excellence
PHE: Public Health England
RR: Risk ratio
SARS-CoV-2: Severe acute respiratory syndrome coronavirus 2
