# Supplementary material for: Comparison of two different frailty measurements and risk of hospitalisation or death from COVID-19: findings from UK Biobank
Source: BMC Med. 2020 Nov 10;18:355. doi: 10.1186/s12916-020-01822-4 (PMC7652674; doi:10.1186/s12916-020-01822-4)
Supplement: Supplementary file 1 — Additional file 1: Table S1. Characteristics of the population according to their COVID-19 test and the frailty index. Table S2. Associations between the frailty phenotype and severe COVID-19 infection (sensitivity analysis). [file 12916_2020_1822_MOESM1_ESM.docx]

**Additional File 1: Comparison of two different frailty measurements and risk of hospitalisation or death from COVID-19: findings from UK Biobank**

**Table S1. Characteristics of the population according to their COVID-19 test and the frailty index.**

|  | **No COVID-19 associated admission or death** | | | **Severe COVID-19 infection** | | |
| --- | --- | --- | --- | --- | --- | --- |
|  | **Fit** | **Mild frailty** | **Moderate/severe frailty** | **Fit** | **Mild frailty** | **Moderate/severe frailty** |
| Total, n | 248,242 | 118,932 | 15,869 (100) | 390 | 323 | 89 |
| Baseline age (years), mean (SD) | 55.7 (8.1) | 57.1 (7.9) | 57.8 (7.6) | 59.5 (8.2) | 60.5 (7.4) | 60.2 (7.0) |
| Current age (years), mean (SD) | 66.8 (8.1) | 68.2 (7.9) | 68.9 (7.6) | 70.6 (8.2) | 71.5 (7.4) | 71.2 (7.2) |
| Sex (female), n (%) | 131,844 (53.1) | 69,360 (58.3) | 9,810 (61.8) | 127 (32.6) | 127 (39.3) | 42 (47.2) |
| Deprivation, n (%) |  |  |  |  |  |  |
| Lower | 88,378 (35.6) | 36,042 (30.3) | 3,367 (21.2) | 102 (26.1) | 73 (22.6) | 13 (14.6) |
| Middle | 84,840 (34.2) | 38,614 (32.5) | 4,249 (26.8) | 129 (33.1) | 90 (27.9) | 14 (15.7) |
| Higher | 75,024 (30.2) | 44,276 (37.2) | 8,253 (52.0) | 159 (40.8) | 160 (49.5) | 62 (69.7) |
| Ethnicity, n (%) |  |  |  |  |  |  |
| White | 235,918 (95.0) | 112,319 (94.4) | 14,698 (92.6) | 345 (88.5) | 287 (88.9) | 80 (89.9) |
| Non-white | 12,324 (5.0) | 6,613 (5.6) | 1,171 (7.4) | 45 (11.5) | 36 (11.1) | 9 (10.1) |
| Smoking status, n (%) |  |  |  |  |  |  |
| Never | 145,907 (58.8) | 60,398 (50.8) | 6,855 (43.2) | 179 (45.9) | 127 (39.3) | 30 (33.7) |
| Previous | 81,660 (32.9) | 44,988 (37.8) | 6,356 (40.1) | 160 (41.0) | 162 (50.2) | 39 (43.8) |
| Current | 20,675 (8.3) | 13,546 (11.4) | 2,658 (16.7) | 51 (13.1) | 34 (10.5) | 20 (22.5) |
| Alcohol intake, n (%) |  |  |  |  |  |  |
| Daily or almost daily | 54,536 (22.0) | 23,183 (19.5) | 2,240 (14.1) | 79 (20.3) | 55 (17.0) | 14 (15.7) |
| One to four times a week | 129,649 (52.2) | 54,548 (45.9) | 5,617 (35.4) | 174 (44.6) | 140 (43.3) | 29 (32.6) |
| One to three times a month | 26,273 (10.6) | 14,357 (12.0) | 2,134 (13.4) | 47 (12.0) | 31 (9.6) | 15 (16.9) |
| Never or special occasions | 37,784 (15.2) | 26,844 (22.6) | 5,878 (37.1) | 90 (23.1) | 97 (30.1) | 31 (34.8) |
| Multimorbidity, n (%) |  |  |  |  |  |  |
| None | 120,531 (48.6) | 14,593 (12.3) | 195 (1.2) | 135 (34.6) | 23 (7.1) | 0 (0) |
| 1 | 88,526 (35.7) | 37,145 (31.2) | 1,255 (7.9) | 154 (39.5) | 74 (22.9) | 4 (4.5) |
| 2-3 | 38,175 (15.4) | 57,656 (48.5) | 7,320 (46.1) | 98 (25.1) | 189 (58.5) | 38 (42.7) |
| ≥4 | 1,010 (0.3) | 9,538 (8.0) | 7,099 (44.8) | 3 (0.8) | 37 (11.5) | 47 (52.8) |

Using a previously validated this frailty index, we classified participants as being fit (frailty index <0.12), mildly frail (frailty index 0.12-0.24) or moderate/severely frailty (frailty index >0.24).

SD: standard deviation; n: number

**Table S2. Associations between the frailty phenotype and severe COVID-19 infection (sensitivity analysis).**

| **Model 5** | **RR (95% CI)** |
| --- | --- |
| Robust | 1.00 (Ref.) |
| Pre-frail | 1.35 (1.16; 1.57) |
| Frail | 1.99 (1.51; 2.62) |

Data presented as RRs with their 95% Cis using Poisson regression analyses. Robust individuals were used as the reference group. The sensitivity analysis was adjusted as per Model 4, but additionally included multimorbidity.
